# Supplementary material for: The Evaluation of Glioblastoma Cell Dissociation and Its Influence on Its Behavior
Source: Int J Mol Sci. 2019 Sep 18;20(18):4630. doi: 10.3390/ijms20184630 (PMC6770747; doi:10.3390/ijms20184630)
Supplement: Supplementary file 1 [file ijms-20-04630-s001.pdf]

Etická komise, Fakultní nemocnice Hradec Králové, Sokolská 581, 500 05 Hradec Králové
Ethics Committee, University Hospital Hradec Kralove, Sokolská 581, 500 05 Hradec Králové

STANOVISKO ETICKÉ KOMISE
Opinion of the Ethics Committee

Vážená paní
MUDr. Petra Kašparová, FPh.D.
Fingerlandův ústav patologie
Fakultní nemocnice Hradec Králové

Číslo jednací/Reference number: 201709 S13P

Řešitel/Principal Investigator: MUDr. Petra Kašparová, FPh.D., Fingerlandův ústav patologie, FNHK

Název studie/Full Title of study: Bunecne kultury glioblastomu

Datum doručení žádosti/Date of submission of the Application Form: 05Sep2017

Datum jednání EK + čas/Date and time of Ethics Committee´s session: 07Sep2017 (14.00-17.00)

Vyjádření EK/ Ethics Committe´s opinion:
EK vydává / EC issues

☒ Souhlasné stanovisko/Favourable opinion
☐ Nesouhlasné stanovisko/Unfavourable opinion

University Hospital Hradec Králové
Ethics Committee
Sokolská 581
500 05 Hradec Králové
Czech Republic

Datum/Date: 08Sep2017

MUDr. Jiří Vortel, předseda EK
Signature of Chairperson of the EC

vyřizuje:Ing. Petra Doležalová, tel.: 49 583 3795; E-mail: etikom@fnhk.cz

Seznam členů etické komise/List of the Ethics Committee Members:

Jméno a příjmení<br>First name and surname	Muž/ Žena<br>Male/<br>Female	Odbornost<br>Specialization	Zaměstnanec zřizovatele EK*<br>Ano<br>Yes	 Ne<br>No	Funkce v EK<br>Role in EC	Přítomen<br>Attendance<br>Ano<br>Yes	 Ne<br>No	Hlasoval<br>Voted<br>Ano<br>Yes	 Ne<br>No
Petra Doležalová, Ing.	F	Economist, University Hospital	☒	☐	member	☒	☐	☒	☐
Ivana Dvořáčková, M.A.	F	Vice-Head Nurse, University Hospital	☒	☐	member	☐	☒	☐	☒
Eduard Havel, M.D., PhD.	M	Physician of the Surgery Dept., University Hospital	☒	☐	member	☒	☐	☒	☐
Josef Herink, Assoc.Prof., M.D., PhD.	M	Physician , Dept. of Toxicology	☐	☒	member	☒	☐	☒	☐
Marta Horáková, Ing.	F	Retired	☐	☒	member	☐	☒	☐	☒
Jaromír Hrubecký, M.D.	M	Retired	☐	☒	member	☐	☒	☐	☒
Petr Hůlek, Prof., M.D., PhD.	M	Physician ,The 2nd Dept. of Internal Medicine, University Hospital	☒	☐	member	☐	☒	☐	☒
Štěpán Klásek	M	Diocesan bishop of Hradec Kralove	☐	☒	member	☒	☐	☒	☐
Bohuslav Král, Prof., M.D., PhD.	M	Physician, The 2nd Dept. of Internal Medicine, University Hospital	☒	☐	member	☒	☐	☒	☐
Jaroslava Pečenková	F	Retired	☐	☒	member	☐	☒	☐	☒
Rosvita Ševčíková, M.A.	F	Lawyer	☐	☒	member	☒	☐	☒	☐
Petra Thomson, PhamDr.	F	Pharmacy, University Hospital	☒	☐	member	☐	☒	☐	☒
Hubert Vaníček, M.D., PhD.	M	Physician, Department of Pediatric Medicine, University Hospital	☒	☐	member	☒	☐	☒	☐
Jiří Vortel, M.D.	M	Cardiologist – private physician	☒	☐	chairman	☒	☐	☒	☐
Jiřina Zatloukalová, M.A.	F	Lawyer	☐	☒	member	☒	☐	☒	☐
Petr Žďánský, M.A.	M	Gerontological and Metabolic Department, University Hospital	☒	☐	Vice-Chairperson	☒	☐	☒	☐

(pozn: *Zaměstnanec zřizovatele EK/ Employee of EC appointing authority)

University Hospital Hradec Králové
Ethics Committee
Sokolská 581
500 05 Hradec Králové
Czech Republic

Datum/Date: 07Sep2017

MUDr. Jiří Vortel, předseda EK
Signature of Chairperson of the EC
